# Glassy Carbon: A Promising Material for Micro- and Nanomanufacturing

**DOI:** 10.3390/ma11101857

**Published:** 2018-09-28

**Authors:** Swati Sharma

**Affiliations:** Karlsruhe Institute of Technology, Institute of Microstructure Technology, Hermann-von-Helmholtz-Platz 1, 76334 Eggenstein-Leopoldshafen, Germany; swati.sharma@kit.edu; Tel.: +49-721-608-29317

**Keywords:** glassy carbon, nanomanufacturing, microfabrication, non-graphitizing carbon, pyrolysis, surface modification

## Abstract

When certain polymers are heat-treated beyond their degradation temperature in the absence of oxygen, they pass through a semi-solid phase, followed by the loss of heteroatoms and the formation of a solid carbon material composed of a three-dimensional graphenic network, known as glassy (or glass-like) carbon. The thermochemical decomposition of polymers, or generally of any organic material, is defined as pyrolysis. Glassy carbon is used in various large-scale industrial applications and has proven its versatility in miniaturized devices. In this article, micro and nano-scale glassy carbon devices manufactured by (i) pyrolysis of specialized pre-patterned polymers and (ii) direct machining or etching of glassy carbon, with their respective applications, are reviewed. The prospects of the use of glassy carbon in the next-generation devices based on the material’s history and development, distinct features compared to other elemental carbon forms, and some large-scale processes that paved the way to the state-of-the-art, are evaluated. Selected support techniques such as the methods used for surface modification, and major characterization tools are briefly discussed. Barring historical aspects, this review mainly covers the advances in glassy carbon device research from the last five years (2013–2018). The goal is to provide a common platform to carbon material scientists, micro/nanomanufacturing experts, and microsystem engineers to stimulate glassy carbon device research.

## 1. Introduction

Carbon has undoubtedly dominated the material science and engineering research in the last two decades. Among various carbon forms, graphene and its derivatives are the most extensively studied materials, which have resulted in nearly 200000 publications since 1995 [1]. Some synthetic carbon forms (i.e., obtained by a bottom-up manufacturing approach), such as fullerenes, offer intriguing scientific questions, for example, related to the rehybridization of the π-atomic orbital [2,3]. Others, such as graphene flakes and carbon nanotubes (CNTs), have attracted considerable attention towards their usability in micro and nano devices [4,5]. The most common, scalable approach for fabricating graphene and CNT-based devices involves mixing these materials (in bulk) with a binder or glue, followed by patterning the thus obtained ‘inks’ onto suitable substrates [6,7]. These fabrication techniques are scalable, but are limited to two-dimensional geometries. The desirable characteristic properties of the individual nanocarbon units are often compromised due to their interaction with the binder [8]. Moreover, handling carbon nanomaterials in powder form is challenging due to their extremely light weight and a lack of safety data on the potential health hazards [9].

A different, top-down manufacturing approach for fabricating graphene-rich carbon devices is to pattern a polymer using widespread lithographic techniques, and carbonize these structures using the pyrolysis process [10,11]. In the last two decades, a variety of microfabrication techniques that utilize radiation-induced lithography have been developed for polymer patterning in the micro/nano scale. Notable examples include photolithography [12], two-photon lithography [13,14], nanoimprint lithography [15,16], X-ray lithography [17], and their combinations. These techniques typically entail a patternable polymer (resist) that can be spin-coated or drop-casted onto a suitable substrate (e.g., silicon wafer) and baked to yield a dry film, which is well below its glass-transition temperature (*T*_g_) at the time of the radiation exposure.

Phenol-formaldehyde (PF) resins, which are well-known glassy carbon precursors, are optimum as resist materials, owing to their thermosetting character, availability in various viscosities, and the possibility of incorporating epoxy side groups that facilitate a chemical amplification after the photoacid generation [18,19]. Consequently, several commercially available photoresists, such as SU-8, AZ resists, mr-NIL resists (tradenames) are primarily composed these resins. Other resists include acrylate-based polymers [20,21] and various inorganic materials [22,23,24], and may entail further optimization when used in the nano-scale fabrication [13,15]. Some of these resists would yield none, or extremely small quantities of carbon on pyrolysis, which may have significantly different properties compared to glassy carbon. Even in the case of a known glassy carbon precursor, a detailed evaluation of the material properties prior to device fabrication is essential, since the actual microstructure of the resulting carbon is influenced by various manufacturing and pyrolysis conditions [25,26,27]. Notably, the exact chemical composition of the commercial resists is often patent-bound; only the elementary chemical unit is disclosed, which provides the first cues on the possibility of its carbonization and the nature of the resulting carbon, but may not be sufficient for a complete assessment of the carbonization mechanism. The focus of this article is glassy carbon-based devices, therefore, only those resists that yield this material are of interest. 

In addition to the carbonization of patterned polymers, device-compatible micro/nano structures can be realized by directly machining glassy carbon. Although this material is difficult to machine, methods, such as electrochemical [28] and thermochemical [29] etching, Focused Ion Beam (FIB) milling [30], and laser machining [31] have been employed to pattern it. In this contribution, some recent glassy carbon micro/nano devices fabricated using both strategies are described. Physicochemical properties of nano-scale glassy carbon where the structure size plays a vital role, and thus renders it dissimilar to the bulk manufactured material, are detailed. An attempt is made to classify all polymer-derived carbons, and correctly place glassy carbon among them. Concerns that have experienced competing views from researchers and are still open for discussion, for example, related to the purity of the low-temperature glassy carbon at the nano-scale, as well as and some nomenclature issues, are evaluated. Prior to indulging into the current research on glassy carbon, a brief history of the development of pyrolytic carbons and their early applications is presented below.

## 2. Brief History of Pyrolytic Carbons

In the early 20th century, graphite became a preferred substitute for metals in various applications, such as battery electrodes and lightweight aircraft parts. Graphite was also widely used as the moderator in the nuclear reactors, and as a high-temperature resistant manufacturing material in the space vehicles (e.g., rocket nozzles, nose shields [32]). This increasing commercial demand motivated the research on artificial graphite and graphite-like materials, which could be produced in large quantities and featured a relatively high purity. As a result, thermal treatment of various organic materials, and the properties of the resulting carbon were extensively studied in the early- and mid-20th century [32,33]. These carbon materials were designated ‘pyrolytic carbon’ or ‘pyrolytic graphite’. It was soon established that the carbon obtained from different precursors had measureable differences in terms of both microstructure and properties. Some were similar to polycrystalline graphite (ABABA type crystal structure), while others featured randomly oriented small graphitic crystallites with a larger interlayer separation and misaligned basal planes, and could not be converted into graphite, even at very high temperatures. These were called non-graphitizing carbons [34] (details in Section 4.2), and were primarily studied using X-ray diffraction (XRD) techniques during their early development [35,36]. Glassy carbon belongs to this category, which is methodically explained in Section 4.

## 3. Pyrolysis

The term pyrolysis is not limited to the heat-treatment of polymers, it defines the thermochemical decomposition of *any* organic precursor, including gaseous hydrocarbons (e.g., acetylene [32,37,38]), hydrocarbon-rich oils [39] and various petroleum byproducts [40]. Gas-phase pyrolysis (i.e., thermal cracking of hydrocarbons [41,42]) followed by carbon deposition is generally referred to as the Chemical Vapor Deposition (CVD) of carbonaceous materials in the contemporary literature [43]. Another well-known application of pyrolysis is in the treatment of waste polymers (both synthetic and natural) for biofuel [1,44,45], and biogas [46] production. Here, the heat treatment temperatures can be below 900 °C, and occasionally the environment may even contain oxygen [47,48], depending on what is expected as the end-product. Pyrolysis is also used in the context of metallurgy (e.g., in pyrometallurgy [49]), where the desirable end-product is usually not carbon [50]. In this article, only the pyrolysis utilized for converting polymers into carbon, especially glassy carbon, is discussed. In order to avoid any confusion, the term ‘polymer-derived carbon’ is used where necessary.

When a polymer is heated above its degradation temperature, carbon-heteroatom bonds start to cleave, which is followed by the formation of the new carbon-carbon bonds [25,51,52]. During the early pyrolysis stages, various hydrocarbon radicals are generated with their highest concentration at around 600 °C [52]. After 800 °C, a network of graphene fragments; containing a large fraction of defects as well as chemical impurities, starts to develop [53]. Further heat allows for the annealing of the defects and an increase in the graphene crystallite size (*L*_a_) and stack thickness (*L*_c_). The absence of oxygen minimizes the CO_2_ and CO formation (i.e., burning), however, if there is any (bonded) oxygen in the polymer itself, some oxides are generated. Other pyrolysis products, such as CH_4_ and small hydrocarbons [54], are volatile that are released in the form of bubbles [55].

Various theories exist on the mechanism of the formation of elemental carbon from a decomposed polymer. For example, it has been proposed that the polymer chains serve as the nucleation point for the resulting graphenic structures in the case of synthetic resins; thus, the graphene crystallites (in glassy carbon) are ribbon or fibril-like [56]. Another theory supports the formation of liquid crystals [57], which subsequently condense to yield a graphitic carbon. In a recent study [53], it was established that the polymer fragments feature a variety of shapes and sizes, which are mobile during the initial pyrolysis stages (500–800 °C), and are constantly trying to attain a thermodynamically stable arrangement by separating from, and merging into, each other [53]. There seems to be no specific pattern in the formation of these fragments. This study, however, is specific to a PF resin. Other polymers may display significantly different disintegration patterns and fragment mobility. For example, pyrolysis mechanism of cellulose (also see Section 4.3) is quite different from that of the resins, which has been studied for over a century [58]. Various polyimides, polyvinyl chloride (PVC), pyridine, etc. have their own characteristic pyrolysis products and reaction kinetics. Major factors that influence the nature of the resulting carbon include the chemical composition of the precursor [26,59], structure size [26,60], heating conditions [26,55], forces applied during fabrication [27], and in the case of epoxy resins, the extent of crosslinking [61].

In addition to the experimental research, there have been various theoretical investigations, for example using the Reactive Molecular Dynamics simulations, for understanding the pyrolysis mechanism of specific polymers [62,63,64]. In a study by Desai et al. [62], it was indicated that the primary fragments formed after the decomposition of a PF resin contain atoms from the neighboring polymer chains in addition to the parent molecule. This finding supports the idea that there are no specific patterns or well-defined graphene nucleation points during the initial pyrolysis and carbon-carbon bond formation stages. Nonetheless, it has been confirmed by both theoretical and experimental investigation that the chemical composition of the polymer plays the most important role in determining the nature of the resulting carbon. Therefore, polymer-derived carbons can be segregated to a first approximation based on the nature of the precursor.

## 4. Polymer-Derived Carbon

**Figure 1:**
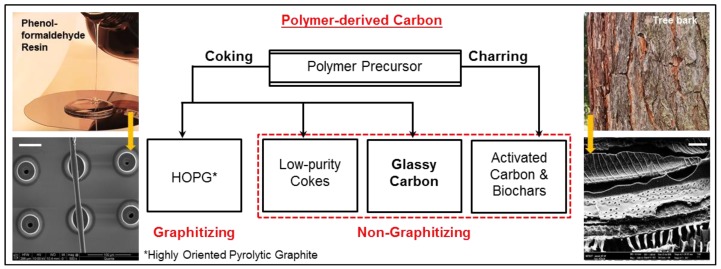
Classification of carbon materials obtained by pyrolysis of polymers. SEM images: Bottom-left: SU-8 micropillars with a suspended SU-8 fiber [65] showing coking (scale bar: 50 µm), and Bottom-right: a tree bark carbonized by charring (scale bar: 10 µm). Both precursors are pyrolyzed at 900 °C at a temperature ramp rate of 5 °C/min in a nitrogen environment.

A classification of polymer-derived carbon based on the precursors is shown in Figure 1, followed by a brief explanation of the terminology.

### 4.1. Coking and Charring

If the pyrolysis product (mixture of all intermediate materials at any given temperature) goes through a semi-solid (rubbery) phase, owing to the fact that its *T*_g_ falls just below the process temperature [21,66], this phenomenon is known as coking [25]. The Scanning Electron Microscope (SEM) image in the bottom-left of Figure 1 represents a carbonized structure where a fiber was intentionally suspended onto an array of hollow micropillars (pillar diameter: ~5x fiber thickness; all structures fabricated in SU-8 [65]). It can be observed that the pillars attached to the fiber are deformed because of the tensile stretching in the fiber during pyrolysis. Other pillars shrink uniformly, indicating that the *T*_g_ of the pyrolysis mixture was only slightly below the set temperature. As this gap increases, the patterned structures further deform [21], and when it is significantly large (e.g., in the case of polyethylene), mostly oil-like materials are obtained [67]. The semi-solid intermediate material is responsible for a smooth surface of the resulting carbon, as it tries to minimize its surface energy. Examples of coking polymers are PF resins (yield non-graphitizing carbon [25]), and anthracene (yields graphitizing carbon [68]).

Charring refers to a direct conversion of the rigid polymer structure into carbon, where the shape is preserved both macro- and microscopically. Wood and other cellulosic polymers are good examples of charring (see the bottom-right SEM image in Figure 1). The major and most studied intermediate formed during cellulose pyrolysis is known as levoglucosan [69,70], which further disintegrates via different pathways leading to the formation of tars (oil-like materials), volatiles and solid carbon [70]. These phases generally coexist and remain distinct throughout the process. The solid carbon backbone is replicated in the final char, and the oils and volatiles are collected and distilled if so desired [1]. Cellulosic materials do encounter some softening in the 230–255 °C region, but it is not directly correlated with either *T*_g_ or the melting point [58]. Chars are predominantly non-graphitizing, and due to their porosity and surface chemistry, often serve as activated carbons. Pyrolysis of natural wood is more complex due to the presence of lignin and hemicellulose [1], which is not detailed here. 

Carbonaceous residues obtained from biodegradable natural polymers at temperatures <900 °C are often called biochars. The term char may also occasionally signify the solid carbon formation from polymers, even after initial coking [25]. Importantly, the nomenclature of carbon materials is already full of ambiguities because of the existence of numerous carbon forms (including those with mixed hybridization), precursors and manufacturing processes. Any terminology that contributes to such confusions should therefore be avoided.

### 4.2. Graphitizing and Non-Graphitizing Carbon 

Graphitizing carbons are those polymer-derived carbons that can potentially be converted into polycrystalline graphite by heat treatment [34,71], catalytic processes [72], stress [73] or any other method. Graphite features an ABABA crystal arrangement with a layer separation of 3.354 Å along the *c*-axis. During initial pyrolysis stages, certain semi-crystalline polymers, such as PVC, convert into a carbonaceous material that resembles stacked graphene fragments. Although initially these fragments contain impurities and have a turbostratic arrangement (randomly rotated basal planes), their progressive ordering at higher temperatures leads towards graphite formation [34,71]. Pyrolytic graphite can be post-processed to yield Highly Oriented Pyrolytic Graphite (HOPG). HOPG can also be obtained using other methods such as recrystallization from the iron melts (commercially known as Kish graphite), or thermal compression of chemically deposited graphene [33].

Non-graphitizing carbons, by definition, cannot be converted into crystalline graphite, as they contain various structural defects, and randomly oriented graphene fragments exhibiting a strong three-dimensional (3D) bonding. These turbostratic graphene fragments feature a Gaussian distribution in terms of size and shape, carbon-carbon bond-length and valance angles [74], and have an inter-layer separation >0.335 nm [25]. The presence of defects causes these fragments to curl, fold and form fullerene-like structures [75,76,77], and occasionally, completely closed buckminsterfullerenes [53]. The curved structures coexist with the larger, stacked graphene sheets [53]. Non-graphitizing carbons exhibit a lower electrical conductivity but an improved hardness compared to graphite, and are also called hard carbons. PF resins, cellulose, poly(vinylidene chloride), and certain polyimides yield non-graphitizing carbon on pyrolysis.

### 4.3. Glassy and Activated Carbon

Non-graphitizing carbon with a high purity that has experienced at least some coking during pyrolysis is known as glassy carbon. In the case of large (millimeter)-scale structures, glassy carbon is obtained at >2000 °C, since the pyrolysis intermediates may display a poor thermal conductivity causing a thermal gradient across the sample [78]. In order to systematically anneal out the volatiles and ensure purity all the way to the center of the structure, these elevated temperatures are essential. In some industrial processes, the preparation of glassy carbon is shown to take place at 1000 °C with modified pyrolysis conditions for ≤3 mm structural dimensions [60]. It has been confirmed by several studies that in the case of micro/nano scale structures, the properties of glassy carbon can be achieved at lower temperatures such as 900 °C (details in Section 5, Section 6 and Section 7), likely with some oxygen impurities.

Activated carbons are non-graphitizing carbons formed by direct charring that contain surface radicals and open pores. They have heteroatoms and more defects compared to glassy carbon, and their electrical conductivity and mechanical strength is typically lower. Owing to an active surface chemistry, they are often used as adsorbants and catalytic beds.

### 4.4. Carbon Fibers

Micro and nano scale fibers prepared by pyrolysis of cellulose, polyacrylonitrile (PAN), or other polymer fibers were traditionally not classified as glassy or activated based on the precursor chemistry [79]. However, with TEM becoming a common characterization tool for carbon materials [80], the microstructure of individual carbon fibers is increasingly being probed, and they are often labeled glassy, graphitic and graphitizing. As such, the annealing pattern in fibers is significantly different from the bulk due to a high surface-to-volume ratio. Surface treatment, fabrication parameters, and in the case of PAN, pre-pyrolysis oxidation can strongly influence their properties [79]. It has been reported that fibers tend to become more ordered (graphitic) if the fabrication process (typically electrospinning [81]) is modified [27], additives are incorporated [82], or stress is introduced [83,84]. Carbon fibers and solely fiber-based structures (e.g., tissue implants) are excluded from this review due to the vastness of the field.

## 5. Glassy Carbon Structures and Devices

As mentioned earlier, glassy carbon features closed porosity due to the presence of fullerene-like structures [76,85], which renders it impermeable to most gases and liquids i.e., chemically inert. Its defect-containing yet stable microstructure is resistant to crack propagation [86]. This property, integrated with a good thermal conductivity, contributes to its high thermal shock resistance. Other general properties of glassy carbon that are interesting from a manufacturing point of view are listed in Table 1. Importantly, the given values are for commercially available (bulk-manufactured) samples, which are typically mentioned in a range rather than as an absolute number, since glassy carbon is not a unique material. For further details regarding the material supplier/grade etc., respective reference articles may be consulted.

In addition to the experimental investigations, properties of glassy carbon under extreme conditions have also been examined theoretically [85]. The mechanical strength of glassy carbon is shown to further improve (at millimeter scale) by its artificial compression [90]. These properties, combined with the possibility of molding the precursor polymer into any desirable shape have been explored for a variety of applications. First, some large-scale applications are detailed, which served as the motivation for micro/nano devices. 

### 5.1. Large-Scale Applications of Glassy Carbon

Glassy carbon has enabled some paradigm shifting technologies such as the fabrication of camera lenses [91] (used for glass molding), development of solid-state batteries (electrode material), and scale-up of harsh chemical processes, such as the crystallization of CaF_2_, CdS and ZnS under extreme P, T conditions (used as vessel liner) [25]. It is used in the manufacture of motor brushes, high temperature furnace elements, and laboratory crucibles previously manufactured using platinum or quartz [25]. Owing to its biocompatibility and mechanical strength, glassy carbon is a recommended material for the replacement of load-bearing joins in the human body [92], and has been studied as a dental implant material [93,94]. Two major challenges associated with the large-scale production of glassy carbon structures are: (i) high costs and (ii) dimensional shrinkage. The cost is directly related to the high processing temperatures, expensive raw materials, and the limitations associated with forming and molding the precursor resins [25]. The second issue, i.e., shrinkage, is inevitable since one must remove all possible non-carbon atoms from the material in order to achieve the useable properties, which leads to an overall mass loss. These challenges are addressed, and even turned into advantages in micro-/nanomanufacturing as described below. 

### 5.2. Glassy Carbon in Micro/Nano Manufacturing

There are two possible pathways for manufacturing micro/nano scale structures using glassy carbon: (i) patterning a polymer followed by pyrolysis for its conversion into glassy carbon, or (ii) direct patterning of glassy carbon in the micro/nano scale. Pathway (i) enables a wider range of shapes without the need for machining, while pathway (ii) has the advantage of known material properties.

#### 5.2.1. Fabrication via Pyrolysis of Polymer Structures

Lithographic techniques for patterning resins are well-studied, and are capable of sub-micron fabrication [16]. When these patterns are pyrolyzed, dimensional shrinkage becomes a tool for further size-reduction, which is otherwise beyond the capabilities of the employed fabrication technique. The thus obtained nano-scale structures offer an enhanced sensitivity and durability, for example, in biosensor applications. Due to a high surface-to-volume ratio of the individual structures, the volatile pyrolysis byproducts are easily annealed out. It has been reported that the material gains a higher crystallinity [27] and a much improved mechanical strength [14] at pyrolysis temperature as low as 900 °C at the nano scale. Selected examples of carbon devices fabricated using this approach from the recent literature are compiled in Table 2. Note that, in some of these articles, the term glassy carbon is not directly used, but the properties of the resulting material are shown to be very close to glassy carbon.

As evidenced by Table 2, the most common material for obtaining micro/nano glassy carbon structures is SU-8 (commercial product from MicroChem, MA, USA). SU-8 is a PF resin (Novolac type), which is extensively used in microfabrication [116], and is typically patterned using standard UV-(photo)lithography for the purpose of carbonization (see entries 1 to 13 in Table 2). UV-lithography is a relatively inexpensive batch fabrication technique [12] that can be optimized to yield <5 µm critical dimensions [104,108]. For large-area patterning of nano-scale structures, photo-nanoimprint lithography [16] is more suitable. This technique can potentially be utilized for obtaining a variety of structured glassy carbon surfaces. SU-8 is also compatible with 3D patterning such as by two-photon lithography [117] and single-photon-multi-layer-interference lithography [118]. However, to my knowledge, carbonization of truly 3D (not 2.5D) SU-8 structures has not been reported. Other polymers used in carbon-based microfarication include IP-resists (commercial product from Nanoscribe GmbH, Eggenstein-Leopoldshafen, Germany) [14,21,111] and various furan resins [112,113,114,115]. Furan resins and polyfurfuryl alcohols are polymers containing the basic furan ring structure [119] that are available in a wide range of viscosities (100 to 300000 centipoise [119]), and have been conventionally used for glassy carbon production. Yet, there are only a few examples of their use in small scale devices, likely due to the fact that they are commonly processed using soft-lithography [114,115] or molding, and entail more processing steps compared to photo-patterning. Microfabrication with furan resins has a lot of scope for further development.

Two major drawbacks of pyrolyzing the substrate-bound, pre-patterned structures for device purposes are (i) non-uniform structural shrinkage and (ii) lack of control over the substrate-carbon interface, occasionally leading to poor adhesion or pattern collapse. After carbonization, the material at the base (footprint) of the structure displays much less *xy*-shrinkage compared to the upper regions. While this effect can be neglected for high-aspect ratio structures, one may end-up with slanted walls as the shape becomes more 2D. Various parameters including the intended geometry, pyrolysis conditions and polymer viscosity influence the pyrolytic shrinkage [120]. 

Good adhesion onto the substrate is crucial for structures that are expected to experience mechanical or fluidic impact during subsequent operation (e.g., glass molding tools [114,115], microfluidic components [105,106] etc.). It can be improved by using adhesion promoters prior to lithography, optimizing the aspect ratio of the structure, and factoring-in the anticipated shrinkage. Direct machining of glassy carbon bypasses the two aforementioned limitations.

#### 5.2.2. Fabrication via Direct Patterning of Glassy Carbon

Representative examples of the direct patterning of glassy carbon are listed in Table 3. Glassy carbon is a hard and brittle material. Therefore, its direct machining using conventional tools is extremely difficult. However, FIB milling [30] and various dry [31] and wet [28] etching methods have been effectively used to pattern it. Its exceptional inertness makes the chemical (wet) etching challenging, but an electrochemically assisted wet etch is generally possible [28], since glassy carbon does exhibit an electrochemical corrosion [121]. Dry etching is often carried out with the aid of reactive ions and/or high-density plasma. FIB milling is an expensive technique for patterning glassy carbon, but one can achieve a critical dimension of 5–10 µm using FIB. Such structures can be used as a master mold in the subsequent fabrication. 

The main challenge associated with direct patterning methods is the resulting surface roughness [122,123]. While glassy carbon surface is characteristically smooth, machining-induced stresses can render it uneven due to its brittleness. Electrochemical wet etching of glassy carbon is capable of yielding submicron features, but its isotropic nature leads to undercuts [123]. Moreover, in the case of etching, regions with a higher crystallinity may have a different etch rate compared to those with defects and imperfections [55]. Laser ablation of glassy carbon suffers from reflections, and the heat generated during the process can potentially lead to altered surface properties [31]. In order to resolve some of these issues, oxidation assisted electrochemical machining [124] has been recently proposed. In this technique, an oxide layer is electrochemically formed (and regenerated) on the surface during its machining, which partly prevents it from getting damaged. 

From Table 2 and Table 3, it is evident that electrode fabrication is one of the most common applications of glassy carbon, which has been extended to some fascinating research topics, such as neural stimulation and recording [95,96,97] and cell separation on microfluidic platforms [105,106]. These applications also benefit from glassy carbon’s biocompatibility, which is substantiated by successful cell culture on glassy carbon platforms [108,109,110].

## 6. Surface Modification of Glassy Carbon Electrodes

Electrodes entail a complete wetting by the electrolyte facilitated by an active surface. Electrode activation can be performed by mechanical polishing, washing, plasma treatment, thermal and laser activation, ultrasonication, and possibly by other methods [132]. This clean and activated electrode is either directly tested, or is subject to further chemical functionalization or electrochemical pretreatment [89,133] prior to its use. This involves an intentional grafting of radicals (e.g., NH^4+^ [134]) or electroactive species (e.g., hydroquinone [135]) specific to the desired application. The surface treatment can be taken one step further by ‘decorating’ the electrodes with other functional materials (e.g., nanoparticles [136], nanotubes [137], other nanocarbon units [138], and their composites [139]), which has been widely performed on glassy carbon. This is an indirect method of using glassy carbon in nano-scale devices, which has led to the development of a variety of electrochemical nano/bio sensors. A detailed analysis of the mechanism of various functionalization pathways can be found in a comprehensive review by McCreery [140].

Catalytic nanoparticles (gold, silver, metal oxides etc.), other carbon nanoforms and their composites may significantly improve the electrocatalytic performance of glassy carbon electrodes [141,142,143]. On the other hand, these nanomaterials benefit from glassy carbon’s high electrochemical and thermal stability [144], enhanced surface area [136], and corrosion resistance. Substrate support improves the available surface area of the nanomaterials, which facilitates a greater mass transport that is of paramount interest in catalysis [145]. Nanoparticles on carbon surfaces are either obtained by reducing metals from their respective salts, or by attaching pre-formed nanoparticles. The adhesion of particles is considerably better in the case of salt reduction, however, pre-synthesized particles allow for a better control over their geometry and other physicochemical properties [146]. Modified electrodes are frequently coated with ionic polymers (e.g., nafion^TM^) to partially block the neutral molecules from reaching the functional element [147].

Selected examples of glassy carbon electrode modification via decoration with other materials are listed in Table 4. The modifying agents are divided into four categories for convenience, and only five examples from each are provided, since the idea here is to present surface modification as a possibility for further sensitivity improvement of miniaturized glassy carbon devices (one such example [107] is listed in Table 2). Notably, in the case of multilayer or composite material coatings, the exact mechanism of the electrocatalytic activity as well as the contribution from glassy carbon itself is occasionally not completely understood. The overall electrode performance, however, is shown to enhance. It is also worth mentioning that the boundaries between surface activation, functionalization, modification and decoration are difficult to set, since every treatment does modify the surface to some extent.

## 7. Characterization of Glassy Carbon

Ensuring that a polymer-derived carbon with an unknown pyrolysis mechanism is glassy carbon is tricky, especially in the micro/nano scale where the first hand visual and physical observations (e.g., glass-like shine) are difficult. Most of the established characterization techniques have specific sample requirements (powders, ultra-thin slices, specialized substrates etc.). In order to satisfy them, one may end up preparing structures of different geometries and surface area compared to what is actually used in the device. Since the annealing mechanism is critical in determining the material purity and porosity [53,60], it is likely to find microstructural variations caused by the sample geometry. To avoid this, one should utilize the techniques that are designed to analyze micro/nano structures where possible, or modify the sample geometry in a way that is not too far from the original. For example, elemental purity can be determined using Energy Dispersive X-ray (EDX) spectroscopy [121], rather than by combustion analysis; or Raman spectroscopy can be performed on an array of microfabricated structures rather than the powder obtained from the same polymer. It is not straightforward to determine whether or not a polymer experiences coking at the micro/nano length scales. One option is to intentionally design a structure that will experience a non-uniform shrinkage during pyrolysis. If it shows bending, twisting or stretching rather than fracturing, this confirms that during the initial pyrolysis stages the material had some viscoelasticity. Importantly, after cooling down, the material becomes hard and brittle in all cases.

Broadly, there are three major aspects of the characterization of polymer-derived carbon: (i) microstructure (to differentiate between graphitizing and non-graphitizing), (ii) surface chemistry and porosity (to establish if it is glassy or activated), and (iii) physicochemical properties (mechanical, electrical, electrochemical) to further narrow down the carbon class. Additionally, elemental purity entails validation in specific cases (for example, when pyrolysis conditions are modified). Commonly used microstructural characterization techniques include XRD [74,164], Raman spectroscopy [164], TEM (preferably low-voltage [53]; can be integrated with Electron Energy Loss Spectroscopy or EELS [165]) and certain physicochemical properties (e.g., electrical conductivity). Surface flatness is generally observed by SEM or AFM measurements, and the chemical nature of the surface is investigated using contact angle measurements (first approximation), and by XPS (detailed surface functional group analysis). Pyrolysis mechanism can be probed using Electron Paramagnetic Resonance [33,52], and the graphitization can be substantiated with magnetoresistance evaluation [38] where possible. All aforementioned techniques complement each other, and may not provide sufficient data as standalone. Further property tests are application-specific.

## 8. Further Discussion Points

### 8.1. Should the Term ‘Glass-Like Carbon’ Be Used Instead of ‘Glassy Carbon’?

According to the IUPAC nomenclature [166], ‘glassy’ and ‘vitreous’ carbon should not be used to denote this material since these terms (i) have been used as trademarks and (ii) give the impression that the material properties are similar to those of amorphous glasses (e.g., silicates), which is incorrect. Despite this recommendation, several pioneering publications and fundamental books used by students and early career researchers contain the term glassy carbon. Subsequently, it is reflected in the Internet search, and is listed as a keyword various publications. Although this is an open question and the choice of the appropriate term is left to the authors, the term glassy carbon is used in this article simply to ensure its wider dissemination.

### 8.2. Is 900 °C a High Enough Temperature to Obtain Glassy Carbon?

One common criticism faced by microsystem engineers is that the carbon obtained below 2000 °C may contain oxygen, and hence, should not be called glassy carbon. Here it is essential to understand that lithographic patterning of polymers entails a substrate, which is typically silicon. The melting point of silicon is 1410 °C however, it may experience thermal fatigue and deform at slightly lower temperatures [167]. Consequently, 900–1200 °C is chosen as the optimum carbonization temperature range for lithography-based devices. It has been frequently reported that the micro/nano carbon structures pyrolyzed at 900 °C feature properties similar to commercial glassy carbons, which is attributed to the high surface-to-volume ratio of the smaller structures that allows them to attain a low porosity and higher purity compared to larger structures at the same temperature. Oxygen impurities, however, are plausible. The exact correlation between structure size, pyrolysis temperature and material properties is a topic for further investigation. A potential solution is to mention the pyrolysis temperature in the label (e.g., glassy carbon-900) in order to avoid ambiguities.

## 9. Conclusions

There are vast global interests in carbon nanomaterials as well as in promoting commercially viable micro- and nanomanufacturing processes. Glassy carbon is an ideal material for this purpose because: (i) it is composed of a stable, percolated graphenic network, is corrosion-free, and hence, remarkably durable at the nano-scale, (ii) it is an elemental carbon form that offers multiple advantages over composites, principally, the availability of all physicochemical properties of the pristine form, (iii) 2.5- and 3D structures can be batch-fabricated by lithographic techniques, (iv) micro-scale techniques can be employed in nanomanufacturing due to the pyrolysis-induced size reduction, and (v) numerous surface modification opportunities without compromising the material properties can be availed. The established large-scale applications of glassy carbon have already proven its commercial viability. Further cost reduction is possible by lowering the required pyrolysis temperature by introducing additives and catalysts in the precursor polymers, development of high performance resins at lower costs, and by moving from batch to continuous manufacturing. There is a substantial scope in the development of direct patterning methods for glassy carbon, for example, by customized etching methods and the use of high-intensity ion and laser beams as direct-writing tools. Owing to its compatibility with 3D microfabrication, this material can potentially be used in a variety of high-aspect ratio devices.

## Figures and Tables

**Table 1:** Properties of commercially available, bulk-manufactured glassy carbons.

Property	Value	Special Conditions, If Applicable	Ref.
Young’s modulus	20–40 GPa	-	[87,88]
Poisson’s ratio	0.15–0.17	-	[87]
Density	1.3–1.55 g/cm^3^	-	[38]
Electrical resistivity	10–50 µΩm	At room temperature	[38]
Thermal expansion coefficient	(2.0–3.4) × 10^−6^ K^−1^	-	[25]
Apparent porosity	0–12%	-	[38]
Electrochemical potential limits (stability window)	(a) 0.9 to −1.1 V	(a) in 1 M HCl	[89]
	(b) 1.4 to −1.5 V	(b) in Phosphate buffer, pH 6	
	(c) 0.5 to −1.6 V	(c) in 1 M NaOH	
	(d) 3.0 to −2.6 V	(d) in 0.2 M LiClO_4_ in acetonitrile	

**Table 2:** Glassy carbon structures for device applications obtained by pyrolysis of micro/nano patterned polymers (representative examples from 2013–2018). Acronyms: IDEA: Interdigitated Electrode Array, NSC: Neural Stem Cell, MRI: Magnetic Resonance Imaging, RF: Resorcinol-Formaldehyde; AFM: Atomic Force Microscopy, PAN: Polyacrylonitrile. Resists are mentioned as Tradenames.

S.No.	Structure/Device	Proposed/Tested Application	Fabrication Technique	Precursor Polymer/Resist	Remarks, If Any	Ref.
1	Microelectrode	Neural sensing	Photolithography	SU-8	Flexible device	[95]
2	Microelectrode	Neural stimulation and recording	Photolithography	SU-8	Flexible device	[96,97]
3	Microelectrode	Dielectrophoresis	Photolithography	SU-8	3D electrodes	[98]
4	Microelectrode	DNA immobilization	Photolithography	SU-8	λ-DNA bridge between electrodes	[99]
5	Microelectrode	Cell sensing	Photolithography	SU-8	Impedance based cell sensing	[100]
6	Microelectrode	Supercapacitor	Photolithography	SU-8	Rapid pyrolysis, bubble containing glassy carbon	[55]
7	Electrode	Heavy metal ion detection	Photolithography	SU-8	Millimeter-scale thin-film electrodes	[101]
8	Microelectrode with suspended nanowires	Gas sensing	Photolithography, electrospinning	SU-8	Device not tested for gas sensing	[102]
9	Microelectrode with suspended nanowires	Chemiresistive biosensor	Photolithography, electrospinning	SU-8	DNA immobilization on carbon nanowire	[103]
10	3D-IDEA	Dopamine sensing (in the presence of ascorbic acid)	Photolithography	SU-8	Redox amplification of dopamine	[104]
11	3D-IDEA	Dielectrophoresis	Photolithography	SU-8	Bacterial analysis, microfluidic device	[105,106]
12	3D-IDEA	Cholesterol sensor	Photolithography	SU-8	IDEAs decorated with gold nanoparticles	[107]
13	Micropillar array	Cell culture (NSCs)	Photolithography	SU-8	-	[108]
14	Porous 3D scaffold	Cell culture (NSCs)	Chemical synthesis, cryogenation	Chitosan, Agarose, Gelatin	Use of MRI for non-invasive characterization	[109]
15	Various cell culture substrates	Cell culture (neuroblastoma and Schwann cells)	Photolithography, electrospinning	RF-gel, SU-8, PAN	Study of cell growth and differentiation	[110]
16	Conical nano-tips	AFM	Two-photon lithography	IP-series resists	Tips printed on silicon cantilevers	[21]
17	Truss	-	Two-photon lithography	IP-series resists	Mechanical property evaluation	[14,111]
18	Micro/nanopillar array	-	Photo-nanoimprint lithography	AR-UL-01	-	[16]
19	Nanoporous thin films	Molecular sieving	Chemical process (dissolution and acetone followed by film deposition)	Polyfurfuryl alcohol	-	[112,113]
20	Inverted microdome	Glass molding	Soft lithography	Furan resin	Master for soft lithography prepared in SU-8	[114]
21	Inverted microfluidic channels	Glass molding	Soft lithography	Furan resin	Microfluidic chip fabrication	[115]

**Table 3:** Devices and structures manufactured by direct patterning of glassy carbon in the micro/nano scale with their applications. Acronyms: RIE: Reactive Ion Etch, FIB: Focused Ion Beam, HOPG: Highly Oriented Pyrolytic Graphite.

S.No.	Structure	Proposed/Tested Application	Patterning Technique	Remarks	Ref.
1	Microtip array	Field emission cathode	Thermochemical etching	Starting material: SU2000 glassy carbon	[29,122]
2	Microtip array	Field emission cathode	Laser machining	Starting material: SU2000 glassy carbon	[125]
3	Test patterns	Fuel cell electrode	Laser machining, reactive ion etching	Comparison/combination of techniques	[31,126]
4	Test patterns (mold for imprinting)	Glass molding for diffractive optical element fabrication	RIE, ion beam etching	RIE under various conditions, comparison of techniques	[127]
5	Test patterns (mold for imprinting)	Glass molding for diffractive optical element fabrication	Inductively Coupled Plasma-RIE	Ti mask used during RIE	[128]
6	Test patterns (mold for imprinting)	Hot embossing of glass	Dicing, laser machining	Starting material: Calcined GC20	[129]
7	Test patterns (mold for imprinting)	Hot embossing/thermal imprinting of glass	FIB milling	Fabrication of microfluidics parts	[30]
8	Test patterns	Electrodes	Electrochemical etching (Anodic in 0.1 M NaOH)	Starting material: photoresist pyrolyzed at 1000 °C	[28,123]
9	Microfluidic channels	Electrospray mass spectrometry	Electrochemical etching (Anodic in 0.1 M NaOH)	Starting material: photoresist pyrolyzed at 1000 °C	[130]
10	Various nanoscale structures	-	Oxygen plasma etching	Hole-mask colloidal lithography used for masking glassy carbon substrate, comparison of glassy carbon and HOPG etch rates	[131]

**Table 4:** Glassy carbon electrodes decorated with functional nanomaterials and their complex mixtures for sensing applications (selected examples from 2013–2018). Acronyms: LOD: Limit of Detection; MWCNT: Multiwalled Carbon Nanotube.

Modifier Type	Modifier Name	Detected Species	Special Features	Ref.
Metals and alloys	Gold nanoparticles	Glucose	Non-enzymatic sensor with a high selectivity; LOD: 0.05 mM	[136]
	Gold-Ruthenium nanoparticles	Indol-3-Carbaldehyde	Detection in the 20–100 μM range; comparison of measurements at different temperatures	[148]
	Silver nanoparticle	Heavy metal ions	Hg+ detected in picomolar concentration	[149]
	Copper nanoparticles	Hydrogen peroxide	Electrodes coated with a thin Nafion^TM^ layer, LOD: 3.45 μM	[150]
	Ni/NiCu alloy films	Mefenamic acid (in Contraflam ^†^)	Electrochemically deposited Ni/NiCu films.	[151]
Metal oxides	Indium-Tin oxide nanoparticles	Sulfides	Selective detection of sulfides in the presence of elemental Sulphur in alcoholic medium, LOD: 0.3 μM	[141]
	Iron oxide (Magnetite and hematite) nanoparticles	Acetaminophen ^†^	Comparison of electrocatalytic activity of glassy carbon electrodes with and without modification	[152]
	SiO2 nanoparticles	Tryptophan ^†^	Detection in real samples, LOD: 5.0 × 10^–8^ mol L^–1^	[153]
	LaCoO_3_ nanostructures	Dopamine, ascorbic acid, uric acid	Simultaneous detection of all species	[154]
	Zinc oxide nanoparticles	Caffeine	Electrochemical detection of Caffeine in tea/coffee samples; LOD: 0.038 μM	[155]
Other carbon forms	Fullerene	Cefitizoxime ^†^	Highly selective determination of Cefitizoxime in a solubilized system; LOD: 0.27 ng/mL	[156]
	Multi-walled CNTs	Valganciclovirn ^†^	LOD: 1.52 × 10^−9^ M (detection in dosage forms), high selectivity	[137]
	Graphene oxide	Nitrobenzene	Comparison of various electrochemical measurement methods. LOD: 66 nM by Cyclic voltammetry.	[157]
	Diamond nanoparticles	Guanine and adenine	Deposition of functionalized diamond nanoparticles embedded in Chitosan; LOD: 2 nM (guanine) and 10 nM (adenine).	[158]
	Nitrogen doped carbon	Caffeic acid in red wines	Use of flame synthesis for preparing N doped carbon; LOD: 0.0024 μM	[159]
Composites and mixtures	Palladium/fullerene	Methane	Electrodeposition of Pd NPs on fullerene films; electrode activity tested at different temperatures	[144]
	Platinum-gold bimetallic nanoclusters/reduced Graphene oxide	Hydrogen peroxide	Nano molar concentrations detected	[160]
	Silver nanoparticles/metal–organic framework composite	Tryptophan ^†^	Metal–organic framework MIL-101(Fe) modified with silver nanoparticles coated onto the electrode; LOD: 0.14 μM	[161]
	Various carbon nanoforms, with and without Li^+^ ions	Heavy metal ions	Comparative study of different modification pathways	[162]
	Metal oxides nanoparticles doped phthalocyanine and functionalized MWCNTs	Dopamine	(MWCNT/Metal Oxide/Phthalocyanine) solution drop casted onto polished electrode; LOD: 0.75 μM	[163]

^†^ Pharmaceutical products.
